# Mental Strain and Chronic Stress among University Students with Symptoms of Irritable Bowel Syndrome

**DOI:** 10.1155/2013/206574

**Published:** 2013-06-16

**Authors:** Marco D. Gulewitsch, Paul Enck, Juliane Schwille-Kiuntke, Katja Weimer, Angelika A. Schlarb

**Affiliations:** ^1^Department of Psychology, Clinical Psychology and Psychotherapy, University of Tübingen, Schleichstraße 4, 72076 Tübingen, Germany; ^2^Department of Internal Medicine VI/Psychosomatic Medicine and Psychotherapy, University Hospital Tübingen, Frondsbergstraße 23, 72076 Tübingen, Germany

## Abstract

*Aim*. To investigate the degree of mental strain and chronic stress in a German community sample of students with IBS-like symptoms. *Methods and Materials*. Following an internet-based survey about stress, this study recruited 176 German university students (23.45 ± 2.48 years; 48.3% males) with IBS-like symptoms according to Rome III and 181 students without IBS (23.55 ± 2.82 years; 50.3% males) and compared them regarding current mental strain (SCL-90-R) and the extend of chronic stress. Beyond this, IBS subtypes, IBS severity, and health care utilization were assessed. *Results*. Students fulfilling IBS criteria showed significantly elevated values of mental strain and chronic stress. Nearly 40% of the IBS group (versus 20% of the controls) reached a clinically relevant value on the SCL-90-R global severity scale. IBS subtypes did not differ in terms of mental distress or chronic stress. Somatization, anxiety, and the chronic stressors “work overload,” “social tension,” and “dissatisfaction with job” were most closely connected to IBS symptom severity. Regarding health care utilization, our results show that consulting a physician frequently was not associated significantly with elevated mental strain or chronic stress but with IBS symptom severity. *Conclusion*. Our data contribute additional evidence to the distinct association between psychological stress and IBS in community samples.

## 1. Introduction

Irritable bowel syndrome (IBS) is a frequent functional gastrointestinal disorder which is characterized by recurrent abdominal pain or discomfort and altered bowel function without an explanatory organic etiology. Despite constipation and/or diarrhea, additional gastrointestinal symptoms like bloating and sensation of incomplete evacuation are experienced quite often.

Epidemiologic data on this disorder vary a lot depending on the examined sample and the diagnostic criteria used. The prevalence of IBS in the general population is estimated to be in the range between 10% and 20% [1–5]. Our previous study has shown that the prevalence of Rome III IBS symptoms among German university students is 18.1% with a significant difference between males (15.2%) and females (21.0%) [6]. IBS, especially in a moderate or severe manifestation, has a considerable impact on health-related quality of life and daily functioning [7–9].

The pathogenic mechanisms of IBS are not fully known. There is strong evidence of altered physiologic features of persons suffering from IBS such as abnormal gastrointestinal motility [10–12] and heightened visceral sensitivity [13–15]. Gastrointestinal infections have also been proposed to predict subsequent IBS [16–19]. Current basic research focuses on the reciprocal connection between symptom-associated physiological and psychosocial features. A key role is ascribed to the interconnections between the central and the enteric nervous system, termed the “brain-gut axis” [20, 21]. Within the framework of this model, a relationship between altered cognitive processes, such as strain and hypervigilance and hence increased arousal of the autonomic nervous system, is linked to increased visceral sensitivity and other IBS symptoms such as modified motility. Vice versa, this leads to an increased amount of perceived stress and symptoms. In this context, chronic stress has been found to be linked to the onset of IBS [22, 23].

A large proportion of persons with IBS have concurrent psychological disturbances. The prevalence of psychiatric disorders ranges from 42% to 94% among patients with IBS in tertiary care [20, 24], but these data may overestimate the actual prevalence of mental health problems, because psychosocial variables can be mediating effects with respect to health care utilisation. This referral bias was reported for the first time in population studies during the 1980s [25, 26]. They observed that individuals suffering from IBS but do not consult physicians, have rates of psychological issues which are comparable to healthy groups. Because of a shortage of up-to-date population-based studies, this fact is still inconclusive [20]. A recent study of Choung et al. [27] found a strong association between psychological factors and IBS in the community. An elevated rate of psychological problems could also be demonstrated in several studies among university or college students with IBS [28–31].

Beside the effect on health care utilization, psychosocial factors may be important in IBS with regard to direct effects on modulation of symptom experience, influence on illness behavior, and impact on choice and outcome of therapeutic interventions [32].

Based on questionnaire data, the current study aimed to investigate the degree of mental strain and chronic stress in a community sample of German university students with IBS-like symptoms in comparison to a non-IBS reference group. The influence of dedicated kinds of stressors and differences between the genders were evaluated and the impact of IBS subtypes and symptom severity on mental health were characterized. In addition, we examined the link between health care utilization, mental strain/chronic stress, and IBS symptom severity.

## 2. Material and Methods

### 2.1. Participants

Participants for this study were recruited during an internet-based survey about stress. In this survey, 425 of 2196 students fulfilled IBS criteria according to Rome III and were invited to complete further questionnaires. A total of 176 participants with IBS (85 males, 91 females) delivered the additional data used in the current study. This subsample did not differ from the whole sample of students with IBS regarding sociodemographic factors or symptom severity. For comparisons, a sex-adjusted control sample (*n* = 181) was recruited from participants not meeting IBS criteria in the previous survey. The control sample was not fully asymptomatic since GI symptoms are common in community [33]. Between the two groups, there were no significant differences in sociodemographic data; for detailed values see Table 1. A comparison of GI symptoms in both groups is also displayed in Table 1. Only two participants reported on a physician-confirmed diagnosis of IBS. Clinically diagnosed mental disorders were mentioned rarely (2.3% and 1.7%).

Participants acknowledging diagnosed gastrointestinal diseases (Crohn's disease, ulcerative colitis, diagnosed lactose or fructose intolerance, celiac disease) were not allocated to the IBS group, neither were women with gastrointestinal complaints only occurring during menses.

### 2.2. Questionnaires

Our definition of IBS was based on Rome III criteria [34]. All data originate from self-report questionnaires. To meet IBS diagnostic criteria, abdominal pain or discomfort had to occur at least 3 days per month in the last 3 months, associated with two or more of the following three symptom features: (i) improvement with defecation, (ii) onset associated with a change in frequency of stool, and (iii) onset associated with a change in form (appearance) of stool. These criteria had to be fulfilled during the last 3 months, and symptom onset had to date back at least 6 months. IBS subtypes were classified as diarrhea predominant (IBS-D), constipation predominant (IBS-C), or alternating (“mixed”) type (IBS-M) according to Rome III criteria [34]. IBS symptom severity was measured by a numeric rating scale ranging from 1 to 10. For some analyses below, the severity was dichotomized into low IBS symptom load (1–5) and high IBS symptom load (6–10). Physician consultations because of the IBS symptoms in the previous three months were assessed by a three-staged answer format (never, once, several times). Subsequent analyses contrasted students with IBS who did not consult a physician (nonconsulters) and students with IBS who saw a physician several times in the last three months (consulters).

The two additional questionnaires used were the German version of the symptom checklist SCL-90-R [35] and the Trier Inventory for the Assessment of Chronic Stress (TICS) [36]. The SCL-90-R [35] is a frequently used questionnaire for the measurement of subjective perception of impairment due to psychological and physical distress. Ninety items are aggregated to nine scales (somatization, compulsiveness, insecurity in social contact, depression, anxiety, aggressiveness/hostility, phobic anxiety, paranoid thinking, and psychoticism). Participants have to rate for each item, to which extent they were bothered by it during the past seven days on a 5-point scale between 0 (not at all) to 4 (very strong). The global severity index (GSI) provides information about the mental strain over the response to all items with higher scores indicating higher mental strain. Many studies have confirmed good psychometric properties, whereas the original factorial structure cannot be replicated in most German samples [37]. The TICS questionnaire [36] asks for stressful experiences within the last three months and consists of 57 items covering the dimensions work overload, worries, social stress, lacking social recognition, work discontent, and intrusive memories. An additional screening subscale of chronic stress (SCSS) consists of 12 items (out of 57). For each item, the frequency of experience within the past 3 months has to be indicated on a 5-point scale from 0 (never) to 4 (very often). Higher values represent greater stress. The TICS features good internal consistency and obtained sound convergent and discriminant validity in subjects between 16 and 70 years [36].

Information about the goals of the study and confirmation of anonymity of the data were provided; informed consent was given deliberately. The study had been approved by the ethics committee of the University Hospital Tübingen.

### 2.3. Data Analysis

All calculations on SCL-90-R and TICS were carried out using the means of the scales (not the standardized *T* values). Standardized *T* values are additionally reported for a better comparability. Group comparisons on the instruments' subscales were performed using two separate MANOVAs to adjust for multivariate effects. Differences between the genders regarding the global indicators GSI and SSCS and differences between IBS subtypes and regarding symptom severity were checked by univariate ANOVAs. (M)ANOVA effect sizes are reported as *η*² (small effect *η*² = 0.01, medium effect *η*² = 0.06, and strong effect *η*² = 0.14).

Considering the strong link between IBS and somatization, further analyses regarding the impact of SCL-90-R subscales on IBS symptom severity were calculated without the scale “somatization.” Stepwise linear regression analyses were used to examine the multivariate influence of SCL-90-R and TICS subscales on IBS symptom severity separately. A joint backward linear regression model including SCL-90-R and TICS subscales was incorporated to predict IBS symptom severity.

## 3. Results

Students suffering from IBS showed significantly higher values on all SCL-90-R subscales and on the global severity index (GSI). The *T*-value differences ranged between 5.8 (somatization) and 2.5 (hostility). The GSI score of IBS students was 5.6 *T*-values above the non-IBS group. Detailed values are shown in Table 2. A proportion of 39.8% of the IBS group reached a clinically relevant *T* value of 60 or above, whereas 20.4% of the non-IBS reference group did,  *χ*² = 15.89, *P* < .001.

Regarding the global GSI, female participants scored higher than males, *F*(1,353) = 16.23, *P* < .001,  *η*² = .44, there was no significant group × gender interaction, *F*(1,353) = 1.06, *P* = .304, Table 3 displays means and standard deviations.

Students fulfilling IBS criteria reported significantly higher values on eight of nine TICS subscales and on the TICS screening scale for chronic stress (TICS-SSCS). Differences between the two groups varied between 6.2 (chronic worries) and 1.1 *T*-values (social overload; n.s.). The TICS-SSCS was 5.3 *T* values higher in the IBS group. Details on comparisons on all subscales are shown in Table 2.

An ANOVA for the TICS-SSCS including the factors group and gender showed a significant effect of gender, *F*(1,353) = 19.07, *P* < .001,  *η*² = .051, with higher values for women and no group × gender interaction, *F*(1,353) = 0.08, *P* = .783. Detailed values are shown in Table 3.

IBS subtypes did not differ with respect to global mental distress (GSI), *F*(1,351) = 0.44, *P* = .643, or chronic stress (TICS-SSCS), *F*(1,351) = 0.38, *P* = .683 (Table 4).

As listed in Table 5, IBS symptom severity went along with an increase in global mental distress (GSI), *F*(2,354) = 28.91, *P* < .001,  *η*² = .140. The severity of IBS symptoms was also accompanied by an increase of chronic stress (TICS-SSCS), *F*(2,354) = 23.32, *P* < .001,  *η*² = .116.

Students satisfying IBS criteria who frequently consulted a physician in the previous three months because of their GI symptoms (*M* = 1.05 ± 0.62; *T* = 60.8) did not differ significantly from nonconsulters with IBS (*M* = 0.80 ± 0.55; *T* = 55.9) in terms of global mental distress (GSI), *t* = −1.88, *P* = .063. This also applied for chronic stress: frequent consulters reached a mean of *M* = 29.09 ± 8.47 (*T* = 64.5) on SSCS which did not differ significantly from non-consulters (*M* = 25.15 ± 9.13; *T* = 60.8), *t* = −1.82, *P* = .071. However, frequent and nonconsulters did differ significantly regarding their reported GI symptom intensity (*M* = 6.14 ± 1.81 versus *M* = 3.74 ± 1.85), *t* = −5.54, *P* < .001.

All SCL-90-R subscales correlated significantly with IBS symptom severity (*r* = .221 to .428), but a linear regression analysis identified only “somatization” (*r* = .428) as a significant predictor of IBS symptom severity (*β* = .428, *t* = 6.27, *P* < .001, adj. *R*² = .179).

A linear regression model of SCL-90-R subscales without “somatization” showed that “anxiety” was the only subscale predicting IBS symptom severity to a significant extend (*β* = .370, *t* = 5.27, *P* < .001, adj. *R*² = .132).

TICS subscales also correlated significantly with IBS symptom severity (*r* = .185 to .263). Stepwise linear regression analysis revealed that “work overload” (*β* = .199, *t* = 2.72, *P* < .01), “social tension” (*β* = .162, *t* = 2.18, *P* < .05), and “dissatisfaction with job” (*β* = .157, *t* = 2.12, *P* < .05) were significant predictors of IBS symptom severity (adj. *R*² = .113).

As shown in Table 6, combined models of “anxiety” (SCL-90-R) and “work overload,” “social tension,” and “dissatisfaction with job” (TICS) resulted in adjusted *R* ^2^s between .149 and .155. Model 3 included only the predictor variables “anxiety” and “work overload” and both reached significance. This minimal model did not differ significantly in explained variance from the other models, which included nonsignificant predictors, and is therefore to prefer.

## 4. Discussion

The aim of the current study was to investigate the degree of mental strain and chronic stress in a sample of young adults suffering from IBS symptoms according to Rome III in comparison to a non-IBS reference group. Additional analyses evaluated the influence of dedicated kinds of stressors and the differences between the genders respective impact of IBS subtypes and symptom severity on mental health and chronic stress.

Whereas subjects with IBS in tertiary care are frequently affected by psychosocial issues, there is ambiguous evidence concerning this association in population-based samples [20, 25–27]. Based on a population-based student sample, our results clearly show significantly elevated values on all SCL-90-R subscales and the global severity index (medium effect size) for the IBS group indicating a generally higher mental strain. This result is in line with a recent study by Choung et al. [27] who also found a strong association between IBS and mental strain by means of SCL-90-R in a community sample. In our study, nearly 40% of the IBS group reached a clinically relevant value on SCL-90-R global severity scale, whereas 20% of the non-IBS group were above the clinical cutoff. Mykletun et al. [38] also reported a considerable comorbidity of IBS and mental disorders. Their study was based on a structured clinical interview in a large community sample and found that half of the population reporting a lifetime IBS diagnosis also had a lifetime mood or anxiety disorder. Studies among samples of university students report consistently higher prevalences of psychological problems in subjects with IBS: Canadian university students with IBS scored higher regarding trait anxiety than asymptomatic controls [28], and a study among Chinese university students highlights the role of anxiety and depression which predicted IBS status [30]. A survey among medical students in Malaysia found that anxiety, depression, insomnia, headache, and backache were encountered more frequently in students with IBS [31].

In our study, students fulfilling IBS criteria reported higher levels of stress on almost all TICS subscales and on the screening scale for chronic stress. Miwa [39] compared community subjects with IBS or dyspepsia with healthy control subjects and specified a significantly higher percentage of IBS subjects who felt stress in their daily lives and a significantly higher percentage of subjects who regarded themselves as being highly susceptible to stress. But it has to be considered that, regarding the possible aetiological role of chronic stress, cross-sectional study designs are not able to provide insights whether psychosocial factors are associated with IBS onset or if they are a consequence of IBS. Using a prospective population-based design, Halder et al. [40] found out that in subjects free of abdominal pain, the factors of psychosocial distress, fatigue, health anxiety, and illness behavior were predictors for a future onset of abdominal pain rather than a consequence. A second study by this workgroup [41] demonstrated that psychosocial distress is an independent risk for the development of IBS in a group of subjects previously free of IBS. Both studies emphasized the impact of somatization and anxiety, which is in line with our results.

As reported before [6] a proportion of 60.4% of surveyed students meeting IBS criteria never consulted a physician because of their GI symptoms. Only two students received an IBS diagnosis by their physician. Several studies showed that most people suffering from IBS are not seen by physicians and thus only a small proportion of patients with IBS gets referred to GI specialists [42–44]. A Malaysian study among medical students demonstrated that only 13.1% of the students with IBS had consulted their health care practitioner [31]. In a landmark study, Drossmann characterized the subsample of consulters to report significantly higher values of psychosocial disturbances [25]. In our sample, this finding could only be replicated to a limited extent in terms of higher numerical values regarding mental strain and chronic stress, but there were no significant differences between the groups. A population-based study by Koloski and colleagues in 2006 [45] did not find psychosocial factors to be discriminating between consulters and nonconsulters either. A study among Canadian university students [28] displayed that students with IBS who sought medical care for IBS exhibited no significant difference in trait anxiety compared to students with IBS who had not consulted a physician. However, consulters were more concerned about the meaning of their symptoms than students who had not sought care. In our study, the subsample of frequent consulters was quite small (*n* = 22) and mental strain and chronic stress are only part of the complex “psychosocial problems.” Other aspects like personality or negative life events may have a strong impact too [32]. One may argue that our sample was restricted to a higher educational background which may be confounded with health care seeking [46].

Studies including chronic stress are often based on a quantification of critical life events in biographical history [47, 48]. Our study clearly shows that ongoing persistent life stress in the last three months is elevated in community subjects with IBS. The psychopathological factors “somatization” and “anxiety” and the chronic stressors “work overload,” “social tension,” and “dissatisfaction with job” are most closely connected to IBS symptom severity. Ruling out “somatization,” the combination of “anxiety” and “work overload” is put out as best predictor combination for IBS symptom severity. The relevance of anxiety is also emphasized by two studies among university and college students in Canada and USA [28, 29] which focused on the role of trait anxiety and anxiety about visceral sensations.

Psychosocial differences between Rome III IBS subtypes in community are not well studied up to now. We found that the three subtypes did not differ with regard to the extent of mental distress or chronic stress. A study by Tillisch et al. found a higher prevalence of psychological symptoms in IBS patients with alternating (mixed) bowel patterns compared to constipation-predominant IBS and diarrhea-predominant IBS patients in tertiary care [49]. Talley et al. could not demonstrate significant group differences on SCL-90-R subscales for constipation-predominant IBS and diarrhea-predominant IBS in tertiary care patients according to Rome-II [50].

A methodological limitation of the current study might be that we assessed IBS symptom severity with a single numeric rating scale, which may possibly be an oversimplification. Following a biopsychosocial approach, severity in IBS might be more than the intensity of pain or other gastrointestinal symptoms. Drossman et al. addressed this issue in their Rome Foundation working team report and suggested that IBS severity is multidimensional and that it is possibly influenced by the intensity of gastrointestinal and extraintestinal symptoms, health-related quality of life, comorbidities, psychosocial factors, degree of functional disability, and illness behavior [51]. Therefore, they proposed a multicomponent rating scale.

Another limitation of this study might be the sampling technique, which recruited participants during an internet-based survey about stress. This approach can be prone to a self-selection bias. It cannot be excluded that especially stressed students were interested in filling in the screening or the additional questionnaires. It is important to notice that self-report questionnaires tend to overdiagnose IBS [52] and no physical examination has been carried out. Beyond that, the current data may only be generalized cautiously to the general population, because of the young age and the higher educational background of our sample.

## 5. Conclusions

Based on a community sample, which was recruited following an internet-based survey about stress, our data contribute additional evidence for the distinct association between psychological factors, namely, mental strain and chronic stress, and IBS in community samples. Health care seeking is not determined by mental strain or chronic stress but associated with IBS symptom severity.

## Figures and Tables

**Table 1:** Sociodemographic data and prevalence of GI symptoms of the IBS group and the non-IBS group.

	IBS group (*n* = 176)	Non-IBS group (*n* = 181)
Gender	85 males (48.3%) 91 females (51.7%)	91 males (50.3%) 90 females (49.7%)
Age	23.45 (±2.48)	23.55 (±2.82)
Number of semesters	5.84 (3.68)	5.84 (3.57)
Flatulence*	60.8%	27.6%
Stomach pain		
Upper abdomen*	23.3%	7.2%
Periumbilical and lower abdomen*	45.5%	11.6%
Defecation		
Diarrhea*	46.0%	5.0%
Obstipation	8.5%	3.9%
Diarrhea and obstipation in turns*	30.7%	5.0%

*Significant difference between the groups (*P* < .001).

IBS: irritable bowel syndrome.

**Table 2:** SCL-90-R and TICS *T*-scores, means, standard deviations, and group comparisons of the IBS group and the non-IBS reference group.

	IBS group (*n* = 176)			Non-IBS group (*n* = 181)			MANOVA			
	*T*	*M*	±	*T*	*M*	±	*F*	df	*P*	*η* ^2^
SCL-90-R										
Somatization	58.22	0.77	0.55	52.44	0.46	0.38	39.17	1, 355	<.001	.099
Obsessive-compulsive	53.75	1.02	0.71	49.61	0.75	0.60	14.20	1, 355	<.001	.038
Interpersonal sensitivity	53.01	0.99	0.78	48.66	0.68	0.58	17.36	1, 355	<.001	.047
Depression	56.71	1.15	0.80	52.27	0.81	0.63	19.07	1, 355	<.001	.051
Anxiety	55.96	0.84	0.77	50.58	0.51	0.56	20.93	1, 355	<.001	.056
Hostility	52.50	0.67	0.70	50.01	0.52	0.61	4.23	1, 355	<.05	.012
Phobic anxiety	54.16	0.35	0.52	50.38	0.20	0.35	10.95	1, 355	<.001	.030
Paranoid ideation	49.80	0.68	0.69	46.76	0.48	0.50	9.90	1, 355	<.001	.027
Psychoticism	54.74	0.51	0.51	51.33	0.34	0.38	12.66	1, 355	<.001	.034
Global severity index (GSI)	56.17	0.82	0.56	50.58	0.55	0.41	25.54	1, 355	<.001	.067
TICS										
Work overload	60.59	19.23	6.51	56.01	16.53	6.79	14.66	1, 355	<.001	.040
Social overload	51.80	9.12	4.92	50.72	8.43	4.49	1.94	1, 355	.165	—
Pressure to succeed	53.37	20.14	5.60	50.86	18.19	5.17	11.67	1, 355	<.01	.032
Dissatisfaction with job	57.14	13.99	5.20	55.25	12.73	5.57	4.87	1, 355	<.05	.014
Excessive work demands	62.44	10.67	4.81	58.55	8.61	4.47	17.51	1, 355	<.001	.047
Lack of social recognition	57.88	7.09	3.31	54.17	5.69	2.89	17.97	1, 355	<.001	.048
Social tension	53.20	7.48	4.80	51.25	6.49	4.17	4.29	1, 355	<.05	.012
Social isolation	60.09	12.34	4.10	57.75	10.56	4.27	15.96	1, 355	<.001	.043
Chronic worries	60.06	10.23	3.84	53.82	7.73	3.86	37.52	1, 355	<.001	.096
Chronic stress (SSCS)	61.06	25.66	8.76	55.79	20.29	8.56	34.34	1, 355	<.001	.088

IBS: irritable bowel syndrome, SCL-90-R: symptom checklist 90 revised, TICS: Trier inventory for the assessment of chronic stress, GSI: global severity index, SSCS: screening scale for chronic stress, MANOVA: multivariate analysis of variance.

**Table 3:** Comparison of global severity index (SCL-90-R) and chronic stress scale (TICS) between IBS group and non-IBS reference group, separated for gender.

	IBS group (*n* = 176)						Non-IBS group (*n* = 181)					
	Men (*n* = 85)			Women (*n* = 91)			Men (*n* = 91)			Women (*n* = 90)		
	*T*	*M*	±	*T*	*M*	±	*T*	*M*	±	*T*	*M*	±
SCL-90-R GSI	54.78	0.68	0.46	57.49	0.94	0.62	49.53	0.48	0.38	51.64	0.63	0.42
TICS SSCS	59.60	23.76	8.71	63.04	27.43	8.48	53.63	18.22	8.25	57.98	22.38	8.40

IBS: irritable bowel syndrome, SCL-90-R: symptom checklist 90 revised, TICS: Trier inventory for the assessment of chronic stress, GSI: global severity index, SSCS: screening scale for chronic stress.

**Table 4:** Comparison of global severity index (SCL-90-R) and chronic stress scale (TICS) between IBS subtypes.

	IBS-D (*n* = 79)			IBS-C (*n* = 14)			IBS-M (*n* = 54)		
	*T*	*M*	±	*T*	*M*	±	*T*	*M*	±
SCL-90-R GSI	56.16	0.81	0.58	54.64	0.77	0.53	57.80	0.89	0.52
TICS SSCS	62.13	26.24	9.03	59.43	24.14	8.16	62.04	26.35	8.49

IBS: irritable bowel syndrome, SCL-90-R: symptom checklist 90 revised, TICS: Trier inventory for the assessment of chronic stress, GSI: global severity index, SSCS: screening scale for chronic stress.

**Table 5:** Comparison of global severity index (SCL-90-R) and chronic stress scale (TICS) between students not meeting IBS criteria and students with IBS (low or high severity).

		No IBS				Low IBS				High IBS			
		*T*	*M*	±	*N*	*T*	*M*	±	*N*	*T*	*M*	±	*N*
SCL-90-R GSI	Men	49.53	0.48	0.38	91	53.71	0.62	0.41	66	58.47	0.91	0.58	19
	Women	51.64	0.63	0.42	90	54.84	0.79	0.53	68	65.35	1.38	0.69	23

TICS SSCS	Men	53.63	18.22	8.25	91	58.64	22.73	8.06	66	62.95	27.37	10.09	19
	Women	57.98	22.38	8.40	90	61.69	26.12	8.23	68	67.04	31.30	8.15	23

IBS: irritable bowel syndrome, SCL-90-R: Symptom checklist 90 revised, TICS: Trier inventory for the assessment of chronic stress, GSI: global severity index, SSCS: screening scale for chronic stress.

**Table 6:** Backward linear regression models for predicting IBS symptom severity.

Model	Predictor variables	*β*	*t*	*P*
1	Anxiety (SCL-90-R)	.255	3.10	<.05
	Work overload (TICS)	.144	1.95	.053
	Dissatisfaction with job (TICS)	.100	1.35	.180
	Social tension (TICS)	.079	1.01	.312
	*F*(4,176) = 9.09, *P* < .001; adj. *R* ^2^ = .155			

2	Anxiety (SCL-90-R)	.284	3.68	<.001
	Work overload (TICS)	.150	2.04	<.05
	Dissatisfaction with job (TICS)	.109	1.47	.143
	*F*(3,176) = 11.77, *P* < .001; adj. *R* ^2^ = .155			
	adj. Δ*R* ^2^ = −.005, *F*_change_(1,172) = 1.03, *P* = .312			

3	Anxiety (SCL-90-R)	.319	4.30	<.001
	Work overload (TICS)	.157	2.14	<.05
	*F*(2, 176) = 16.47, *P* < .001; adj. *R* ^2^ = .149			
	adj. Δ*R* ^2^ = −.010, *F*_change_(1,173) = 2.17, *P* = .143			

IBS: irritable bowel syndrome, SCL-90-R: symptom checklist 90 revised, TICS: Trier inventory for the assessment of chronic stress.
